# Glial Sulfatides and Neuronal Complex Gangliosides Are Functionally Interdependent in Maintaining Myelinating Axon Integrity

**DOI:** 10.1523/JNEUROSCI.2095-18.2018

**Published:** 2019-01-02

**Authors:** Rhona McGonigal, Jennifer A. Barrie, Denggao Yao, Mark McLaughlin, Madeleine E. Cunningham, Edward G. Rowan, Hugh J. Willison

**Affiliations:** ^1^University of Glasgow, Institute of Infection, Immunity and Inflammation Glasgow G12 8TA, United Kingdom,; ^2^University of Glasgow, School of Veterinary Biosciences, Glasgow G61 1QH, United Kingdom, and; ^3^University of Strathclyde, Strathclyde Institute of Pharmacy and Biochemical Sciences, Glasgow G4 0RE, United Kingdom

**Keywords:** axo–glial integrity, ganglioside, MAG, NF155, node of Ranvier, sulfatide

## Abstract

Sulfatides and gangliosides are raft-associated glycolipids essential for maintaining myelinated nerve integrity. Mice deficient in sulfatide (cerebroside sulfotransferase knock-out, *CST*^−/−^) or complex gangliosides (β-1,4-*N*-acetylegalactosaminyltransferase1 knock-out, *GalNAc-T*^−/−^) display prominent disorganization of proteins at the node of Ranvier (NoR) in early life and age-dependent neurodegeneration. Loss of neuronal rather than glial complex gangliosides underpins the *GalNAc-T*^−/−^ phenotype, as shown by neuron- or glial-specific rescue, whereas sulfatide is principally expressed and functional in glial membranes. The similarities in NoR phenotype of *CST*^−/−^, *GalNAc-T*^−/−^, and axo–glial protein-deficient mice suggests that these glycolipids stabilize membrane proteins including neurofascin155 (NF155) and myelin-associated glycoprotein (MAG) at axo–glial junctions. To assess the functional interactions between sulfatide and gangliosides, *CST*^−/−^ and *GalNAc-T*^−/−^ genotypes were interbred. *CST*^−/−^× *GalNAc-T*^−/−^ mice develop normally to postnatal day 10 (P10), but all die between P20 and P25, coinciding with peak myelination. Ultrastructural, immunohistological, and biochemical analysis of either sex revealed widespread axonal degeneration and disruption to the axo–glial junction at the NoR. In addition to sulfatide-dependent loss of NF155, *CST*^−/−^ × *GalNAc-T*^−/−^ mice exhibited a major reduction in MAG protein levels in CNS myelin compared with WT and single-lipid-deficient mice. The *CST*^−/−^ × *GalNAc-T*^−/−^ phenotype was fully restored to that of *CST*^−/−^ mice by neuron-specific expression of complex gangliosides, but not by their glial-specific expression nor by the global expression of *a*-series gangliosides. These data indicate that sulfatide and complex *b*-series gangliosides on the glial and neuronal membranes, respectively, act in concert to promote NF155 and MAG in maintaining the stable axo–glial interactions essential for normal nerve function.

**SIGNIFICANCE STATEMENT** Sulfatides and complex gangliosides are membrane glycolipids with important roles in maintaining nervous system integrity. Node of Ranvier maintenance in particular requires stable compartmentalization of multiple membrane proteins. The axo–glial adhesion molecules neurofascin155 (NF155) and myelin-associated glycoprotein (MAG) require membrane microdomains containing either sulfatides or complex gangliosides to localize and function effectively. The cooperative roles of these microdomains and associated proteins are unknown. Here, we show vital interdependent roles for sulfatides and complex gangliosides because double (but not single) deficiency causes a rapidly lethal phenotype at an early age. These findings suggest that sulfatides and complex gangliosides on opposing axo–glial membranes are responsible for essential tethering of the axo–glial junction proteins NF155 and MAG, which interact to maintain the nodal complex.

## Introduction

**Figure 1.**
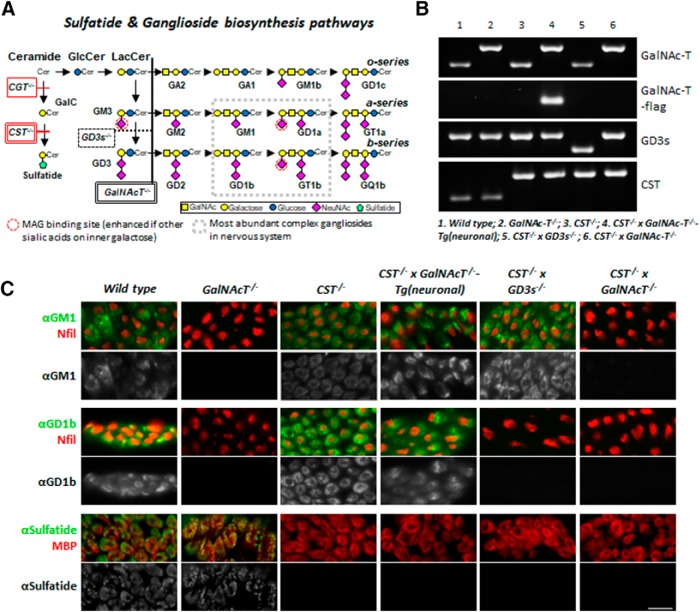
Generation of lipid-deficient transgenic mouse lines and confirmation by PCR and immunostaining. Six mouse lines were used and generated: WT, *GalNAc-T*^−/−^, *CST*^−/−^, *CST*^−/−^ × *GalNAc-T*^−/−^-Tg(neuronal), *CST*^−/−^ × *GD3s*^−/−^, and *CST*^−/−^ × *GalNAc-T*^−/−^. ***A***, Sulfatide and ganglioside biosynthesis pathways (associated gene knock-outs are indicated in boxes). Ceramide is the precursor to sulfatide and gangliosides. The CST enzyme is necessary for the synthesis of sulfatide from GalC. The GalNAc-T enzyme is necessary for generation of complex gangliosides and the GD3s enzyme for specific production of *b*-series complex gangliosides. Constructs were generated to drive GalNAc-T expression in neurons of *GalNAc-T*^−/−^ × *CST*^−/−^ mice to produce the *GalNAc-T*^−/−^-Tg(neuronal) mouse line. Location of MAG binding site to terminal 2,3 sialic acid is indicated by a red circle. ***B***, PCR results confirming the presence or absence of GalNAc-T, GD3s, and CST expression in mouse lines. Large bands represent the disrupted GalNAc-T and CST gene with insert; the smaller band represents the disruption of the GD3s gene. The flag identifies the reintroduction of the GalNAc-T gene into the neurons. ***C***, Expression of *a*- and *b*-series gangliosides and sulfatide was confirmed by staining peripheral nerves with anti-GM1, anti-GD1b, and anti-sulfatide monoclonal antibodies (green), respectively. Axons and myelin were identified with neurofilament or MBP (red), respectively. Scale bar, 10 μm.

Gangliosides are enriched in lipid rafts (Simons and Toomre, 2000) involved in the topographical organization of membrane proteins (Jackman et al., 2009; Sonnino et al., 2014, 2015). Complex ganglioside biosynthesis requires β-1,4-*N*-acetylegalactosaminyltransferase 1 (*GalNAc-T*) enzyme activity (see Fig. 1*A*) with ganglioside expression being widespread in both neuronal and glial membranes. Sulfatide, 3-O-sulfogalactosylceramide, synthesized from galactocerebroside (GalC) by cerebroside sulfotransferase enzyme (*CST*) (see Fig. 1*A*), is enriched in the outer leaflet of the myelin and uncompacted glial membranes (Ishizuka, 1997) and small amounts are present in neurons and astrocytes (Eckhardt, 2008). Evidence indicates that both sulfatide and complex gangliosides are crucial for the maintenance and stability of, rather than the developmental formation of, nervous system domains (Takamiya et al., 1996; Honke et al., 2002; Sturgill et al., 2012). Mice deficient in these lipids thus develop relatively normally before showing age-dependent degeneration (Takamiya et al., 1996; Honke et al., 2002). Evidence indicates that these glycolipids are essential for stabilizing the normal arrangement of axo–glial interactions, particularly at the paranodal region of the node of Ranvier (NoR). Mice deficient for GalC and sulfatide (Dupree et al., 1999), sulfatide alone (Ishibashi et al., 2002; Marcus et al., 2006; Hoshi et al., 2007), or complex gangliosides (Susuki et al., 2007) have prominently disrupted paranodes. The paranode in both the PNS and CNS tethers and seals the abutted axon and glial membranes between the node and juxtaparanode (Garcia-Fresco et al., 2006; Susuki et al., 2013), thereby maintaining voltage-gated nodal sodium (Nav) and juxtaparanodal potassium (Kv) channel clustering and segregation, which are essential for normal conduction (Salzer, 1997; Poliak and Peles, 2003). The importance of appropriate expression and localization of the paranodal proteins in stabilizing Nav channel clusters is also revealed in paranodal protein knock-out mice. Therefore, deficiency in contactin, contactin-associated protein (Caspr), or neurofascin 155 (NF155) leads to progressive paranodal defects from postnatal day 10 (P10) and subsequent axon degeneration, with most mice dying by P21 (Bhat et al., 2001; Boyle et al., 2001; Pillai et al., 2009). Significantly, whereas these adhesion complex proteins comprise the physical adhesion barrier at the paranode, associated glycolipid molecules appear to be required for their localization and stabilization, likely by the targeting and transportation of adhesion molecules in lipid rafts. In support of this, Schafer et al. (2004) showed a reduction in raft-associated NF155 and paranodal localization in sulfatide and GalC deficiency, thereby limiting its co-clustering function with axo–glial partners. Additionally, extraction studies showed that sulfatide-containing lipid rafts as anchors for the axo–glial adhesion molecule myelin-associated glycoprotein (MAG), as well as NF155 (Pomicter et al., 2013).

Complex gangliosides are also an important lipid raft component required for paranode stabilization, as shown by disorganization of paranodal proteins in *GalNAc-T*^−/−^ mice (Susuki et al., 2007). Because gangliosides are present in both neuronal and glial membranes, their principal site of action in paranodal stabilization cannot be presumed. To address this, we demonstrated that loss of neuronal complex gangliosides underpins the *GalNAc-T*^−/−^ phenotype because selective reintroduction of complex ganglioside expression in neuronal but not glial membranes rescues the paranodal phenotype (Yao et al., 2014). Complex gangliosides GT1b and GD1a, both prominently expressed on the axonal membrane, are *trans*-receptors for glial membrane-associated MAG (see Fig. 1*A*) (Collins et al., 1997; Vinson et al., 2001). Analysis of protein extracts demonstrated a reduction in MAG levels in *GalNAc-T*^−/−^ mice (Sheikh et al., 1999; Kawai et al., 2001), which suggests that complex neuronal gangliosides and glial MAG cooperatively contribute to the stability of the axo–glial junction.

Importantly, loss of sulfatide does not alter ganglioside content (Honke et al., 2002) and complex ganglioside deficiency does not alter sulfatide content (Yamashita et al., 2005), which suggests that their expression is not directly interlinked in a compensatory way, but nevertheless might be additive functionally. Here, we considered that, because these glycolipids are differentially expressed in glial and axonal membranes, they may act in partnership to retain clustered proteins in their respective domains. We sought to test this by interbreeding strains of single-null mice and neuronal- and glial-specific rescue mice, thereby allowing us to assess interdependency and cooperativity in the role of axonal and glial glycolipids in paranodal organization.

## Materials and Methods

### 

#### Mice

Seven mouse lines on the C57BL/6 background were used and generated: (1) WT; (2) *GalNAc-T*^−/−^; (3) *CST*^−/−^; (4) *CST*^−/−^ × *GalNAc-T*^−/−^-Tg(neuronal); (5) *CST*^−/−^ × *GD3s*^−/−^; (6) *CST*^−/−^ × *GalNAc-T*^−/−^; and (7) *CST*^−/−^ × *GalNAc-T*^−/−^-Tg(glial) (group number and age are described per experiment below). Generation of *GalNAc-T*^−/−^ and *CST*^−/−^ transgenic mice has been described previously (Takamiya et al., 1996; Honke et al., 2002) and were interbred to produce the *CST*^−/−^ × *GalNAc-T*^−/−^ genotype. Double-null mice with reconstituted site-specific expression of complex gangliosides were produced by crossing the *CST*^−/−^ genotype with previously described *GalNAc-T*^−/−^-Tg(neuronal) or *GalNAc-T*^−/−^-Tg(glial) strains (Yao et al., 2014) or *GD3s*^−/−^ (Okada et al., 2002), resulting in *CST*^−/−^ × *GalNAc-T*^−/−^-Tg(neuronal), *CST*^−/−^ × *GalNAc-T*^−/−^-Tg(glial), and *CST*^−/−^ × *GD3s*^−/−^, respectively. *CST*^−/−^ × *GalNAc-T*^−/−^-Tg(glial) bred poorly and there were insufficient numbers to fully phenotype, so their inclusion was restricted to survival data and phenotypic analysis. Mice were maintained under a 12 h light/dark cycle in controlled temperature and humidity with *ad libitum* access to food and water. For each study, mice of either sex were killed by rising CO_2_ inhalation; all experiments using mice were performed in accordance with a license approved and granted by the United Kingdom Home Office and conformed to University of Glasgow institutional guidelines. Experiments complied with relevant guidelines on the care and use of animals outlined in the revised Animals (Scientific Procedures) Act of 1986.

#### Phenotypic analysis of mice

Weights were obtained at P22 from WT (*n* = 5), *GalNAc-T*^−/−^ (*n* = 5), *CST*^−/−^ (*n* = 4), *CST*^−/−^ × *GalNAc-T*^−/−^-Tg(neuronal) (*n* = 4), *CST*^−/−^ × *GD3s*^−/−^ (*n* = 6), and *CST*^−/−^ × *GalNAc-T*^−/−^ (*n* = 10) mice. Data points from every animal per genotype were plotted and the mean and SEM displayed. Survival plots for all genotypes were plotted over 200 d for each genotype: WT (*n* = 7); *GalNAc-T*^−/−^ (*n* = 10); *CST*^−/−^ (*n* = 18); *CST*^−/−^ × *GalNAc-T*^−/−^-Tg(neuronal) (*n* = 6); *CST*^−/−^ × *GD3s*^−/−^ (*n* = 38); *CST*^−/−^ × *GalNAc-T*^−/−^-Tg(glial) mice (*n* = 12); and *CST*^−/−^ × *GalNAc-T*^−/−^ (*n* = 29). Mice were photographed when suspended by the tail to record hind-limb leg splaying (a feature of many neurodegenerative mutants) and gross brain anatomy was also recorded upon brain removal at P22.

#### Materials

Monoclonal antibodies used to detect complex *a*- or *b*-series gangliosides by immunofluorescent staining were generated and described previously (Bowes et al., 2002; Boffey et al., 2005). Anti-GM1 ganglioside antibody (DG2) and anti-GD1b antibody (MOG1) were used at 20 μg/ml. A new monoclonal anti-sulfatide antibody (GAMEG3) derived from mice inoculated with sulfatide-bearing liposomes was used to detect sulfatide (Meehan et al., 2018). The following primary antibodies were used: mouse anti-phosphorylated neurofilament-H antibody (NF-H, BioLegend; RRID:AB_2715851; 1:2000); rat anti-MBP (Bio-Rad; RRID:AB_325004; 1:500); mouse anti-pan Nav (Sigma-Aldrich; RRID:AB_477552; 1:100); mouse anti-ankyrin G (Thermo Fisher Scientific; RRID:AB_2533145; 1:100); rabbit anti-Caspr (gifted from Professor Peles, Weizmann Institute, Israel; 1:1500); rabbit anti-Kv1.1 (Alomone Laboratories; RRID:AB_2040144; 1:200); rabbit anti-Nav1.6 (Sigma-Aldrich; RRID:AB_477480; 1:100); rabbit anti-pan neurofascin (anti-pNFasc; gifted from Professor Brophy, University of Edinburgh, UK; 1:1000); and mouse anti-MAG antibody (gifted from Professor Brophy, University of Edinburgh, UK; 1:100). Primary antibodies used in Western blots were rabbit anti-MAG 248 (gifted from Prof. N Groome, 1:10000) (Barrie et al., 2010) and rabbit anti-NF155 (gifted from Professor Brophy, University of Edinburgh, UK; 1:5000). Secondary antibodies for immunofluorescent staining were prepared in PBS plus 1% NGS: isotype-specific (IgG1, IgG3) Alexa Fluor 488- and Alexa Fluor 555-conjugated goat anti-mouse IgG antibodies (Invitrogen; RRID:AB_141780); Alexa Fluor 488- and Alexa Fluor 555-conjugated goat anti-rabbit (Invitrogen; RRID:AB_141761); and anti-rat IgG antibodies (Invitrogen; RRID:AB_141733). Secondary antibody for Western blots was HRP-linked goat anti-rabbit (Dako, 1:10,000) prepared in 5% skim milk/TBS containing 0.1% Tween 20 (T-TBS).

#### Immunostaining, image acquisition, and analysis

##### Lipid localization.

Snap-frozen peripheral nerves were transversely sectioned at 10 μm and collected onto APES-coated slides (*n* = 3/genotype). Sections were treated with 100% EtOH for 10 min at −20°C and then thoroughly washed in PBS. Antibodies prepared in PBS were applied to the sections overnight at 4°C in the following combinations anti-ganglioside antibody DG2 or MOG1 with anti-phosphorylated NF-H antibody and anti-sulfatide antibody with anti-MBP antibody. Sections were washed in PBS and secondary antibodies applied for 1 h at room temperature, washed in PBS, and mounted in Citifluor. Representative images were captured at 40× magnification using a Zeiss AxioImager Z1 with ApoTome attachment and processed using Zeiss Zen 2 blue edition software.

##### Nodal protein immunostaining.

Sciatic nerves (SNs) and optic nerves (OpNs) were fixed for 30 min in 4% PFA upon removal from P22 mice (*n* = 3/4 per genotype). Nerves were cryoprotected in 30% sucrose and either gently teased into single fibers (SNs) collected on slides or frozen in optimal cutting temperature mounting medium and longitudinally sectioned at 10 μm (OpNs). To study nodal protein localization, teased SNs were immunostained with primary antibodies for anti-Nav1.6, anti-Kv1.1, or pNFasc and OpN sections were immunostained with primary antibodies for combinations of anti-pNav and anti-pNFasc or anti-Caspr and anti-AnkG. OpN sections and unfixed peripheral nerve sections were stained with anti-MAG antibody. Nerves were pretreated with blocking solution (10% NGS + 0.3% Triton X-100) for 1 h at 4°C before incubation overnight in the same solution plus primary antibody combinations at the same temperature. Triton X-100 was omitted from blocking and incubation solutions when using unfixed peripheral nerve sections. Samples were washed 3× for 5 min and then incubated for 2 h at room temperature in secondary antibody solution. After 3 further 5 min PBS washes, slides were mounted in Citifluor. All nodal protein images were captured at 63× magnification and tissue was MAG immunostained at 40× magnification using a Zeiss AxioImager Z1 with ApoTome attachment and processed with Zeiss Zen 2 blue edition software. For teased SNs, 10 ROIs (capturing 1–12 NoRs) were imaged and quantified per mouse for each protein. For OpN double-staining combinations, three 10-μm-thick *z*-stacks (30 slices, step value 0.34 μm) were captured per mouse. Four 50 × 50 μm fields of view (FOV) per stack were analyzed for pNav channel cluster number, number of Nav channel clusters flanked by intact NFasc dimers, or intact Caspr dimers flanking AnkG using ImageJ software (RRID:SCR_003070). Clusters/dimers were included in the count if they overlaid the top and right borders of the FOV and excluded when they overlaid the left or bottom borders. For MAG staining intensity analysis, single slices were captured at set exposures from three slides per genotype. Graphs ± SEM or box-and-whisker plots were used to display the spread of all data points collected from each animal then means were calculated per genotype for statistical analysis.

##### Ultrastructure.

Mice (WT, *n* = 5; *GalNAc-T*^−/−^, *n* = 6; *CST*^−/−^, *n* = 6; *CST*^−/−^ × *GalNAc-T*^−/−^-Tg(neuronal), *n* = 6; *CST*^−/−^ × *GD3s*^−/−^, *n* = 6; and *CST*^−/−^ × *GalNAc-T*^−/−^, *n* = 5) were transcardially perfused at P25 with a 5% glutaraldehyde/4% paraformaldehyde mixture before the OpN was removed and processed for resin embedding, as described previously (Griffiths et al., 1981). Sections were cut for both light and ultrastructural analysis. Electron micrographs from transverse sections of the OpN at 6700× magnification were captured on a Jeol CX-100 electron microscope. For quantification, a minimum of 10 electron micrographs per animal were taken of randomly selected fields. All measurements were made on scanned images using ImageJ software. For axon morphometry and quantification of axonal changes, all axons within or touching the top and left borders of an region of interest (ROI) were counted. The axon density and number of degenerating axons within the ROI were counted. Averaged values from every animal per genotype was plotted and the mean and SEM displayed.

#### Extracellular recordings

Perineural recordings were made from triangularis sterni nerve–muscle preparations set up as described previously (Braga et al., 1991; McGonigal et al., 2010). Recordings were made from small nerve bundles from each genotype (WT, *n* = 5; *GalNAc-T*^−/−^, *n* = 2; *CST*^−/−^, *n* = 3; *CST*^−/−^ × *GalNAc-T*^−/−^, *n* = 2). Perineural Nav and Kv channel waveforms were collected during a paired pulse stimulation protocol. A representative graph plotting the peak Nav and Kv values as a percentage of the baseline waveforms at each interstimulus interval (ISI) was used to convey recovery of the nodal ion channel currents. A two-way ANOVA was used to compare the difference between genotypes and *post hoc* multiple comparisons to measure differences at each ISI.

Conduction velocity was measured in SN (WT, *n* = 3; *GalNAc-T*^−/−^, *n* = 4; *CST*^−/−^, *n* = 4; and *CST*^−/−^ × *GalNAc-T*^−/−^, *n* = 5), as described previously (McGonigal et al., 2010). OpNs (between the eyeball and the optic chiasm) were quickly removed into oxygenated (95% O_2_ and 5% CO_2_) physiological Ringer's solution containing the following (in mm): NaCl, 129; KCl, 3; NaH_2_PO_4_, 1.2; CaCl2, 2.4; MgSO_4_ 1.3; HEPES, 3; NaHCO3, 20; and glucose, 10. The nerves were mounted in a recording chamber and each end drawn into suction electrodes for electrophysiological studies (WT, *n* = 4; *GalNAc-T*^−/−^, *n* = 4; *CST*^−/−^, *n* = 4; *CST*^−/−^ × *GalNAc-T*^−/−^-Tg(neuronal), *n* = 3; *CST*^−/−^ × *GD3s*^−/−^, *n* = 6; and *CST*^−/−^ × *GalNAc-T*^−/−^, *n* = 3). Compound action potentials were evoked by a supramaximal stimulus (Grass S88 stimulator), applied via a suction electrode at the proximal cut end, and recorded from a second suction electrode at the distal cut end. Signals were amplified (CED1902), digitized (NIDAQ-MX A/D converter; National Instruments), and analyzed using WinWCP version 4.1.0 (J. Dempster, Strathclyde University, RRID:SCR_014713). Nerves were crushed at the end of the experiment to identify and exclude electrical artifacts from the analysis. Nerve conduction velocity was calculated by dividing the length of the nerve by latency (the time between the stimulation artifact and the highest peak of compound action potential). Average values from every animal per genotype were plotted and the mean and SEM displayed.

#### Western blot

Mouse brains were removed (*n* = 4/genotype), snap frozen in liquid nitrogen, and stored at −80°C until required. Myelin preparation was conducted using a modification of the method by Norton and Poduslo (1973). Briefly, brains were homogenized in a buffer composed of 0.85 m sucrose, 10 mm HEPES, pH 7.4, 2 mm DTT, and 1 mm TLCK for 20 s using an Ultra-Turrax T8 blender (IKA-Works) set at maximum speed and 0.25 m sucrose gently layered on top of the homogenate and then centrifuged at 70,000 × *g* for 90 min at 4°C. The myelin interface was collected, hypotonically lysed in chilled dH_2_O, and pelleted at 23,000 × *g* for 30 min at 4°C. Following an additional two rounds of hypotonic lysis, the myelin pellet was resuspended in 10 mm HEPES, pH 7.4, containing 1× protease inhibitor mixture (Sigma-Aldrich). The myelin fraction was stored at −80°C until required. The protein concentration was determined using a bicinchoninic acid method using BSA as a standard (Pierce). SDS-PAGE/ Western blot analysis was conducted as described previously (Yool et al., 2001). In brief, 1 and 5 μg of myelin was denatured in Laemmli buffer, separated on a 4–12% gel (Bio-Rad), and transferred to nitrocellulose membrane (Invitrogen). The membrane was blocked with 5% skimmed milk in T-TBS for 1 h at room temperature and then incubated in primary antibodies prepared in 5% semi-skim milk in T-TBS overnight at 4°C on an orbital shaker. Following three 10 min washes in T-TBS, the immunocomplex was detected with HRP-linked secondary antibody (Dako) and visualized using the ECL reaction as per the manufacturer's instructions (Pierce). Densitometric analysis of the Western blots was performed using ImageJ software. Average values from every animal per genotype were plotted and the mean and SEM displayed.

#### Experimental design and statistical analysis

The numbers of independent animals are described in the Materials and Methods and Results sections and indicated in the figure legends. Statistical differences among genotypes were determined by one-way or two-way ANOVA followed by a Fisher's or Tukey's *post hoc* tests using GraphPad Prism 6 software (RRID:SCR_002798). Differences were considered significant when *p* < 0.05.

## Results

### Ganglioside and sulfatide expression is successfully eliminated in CST^−/−^ × GalNAc-T^−/−^ mice

To investigate the interaction between ganglioside and sulfatide lipids on nervous system integrity, we studied six transgenic mouse lines: WT; *GalNAc-T*^−/−^; *CST*^−/−^; *CST*^−/−^ × *GalNAc-T*^−/−^-Tg(neuronal); *CST*^−/−^ × *GD3s*^−/−^; and *CST*^−/−^ × *GalNAc-T*^−/−^. Table 1 provides an overview of the specific glycolipid expression and deficiency profiles among the mice generated for this study. We confirmed the presence of targeted gene disruption and reintroduction in these transgenic lines by PCR and screening for complex ganglioside or sulfatide expression in neural tissue by immunostaining (Fig. 1). An additional line, *CST*^−/−^ × *GalNAc-T*^−/−^-Tg(glial), was subsequently generated for comparative survival plots. The CST, GalNAc-T, and GD3s genes are disrupted by an insert, which was assessed by PCR. In Figure 1*B*, the larger band represents successful GalNAc-T gene disruption in *GalNAc-T*^−/−^, *CST*^−/−^ × *GalNAc-T*^−/−^-Tg(neuronal) mice, and *CST*^−/−^ × *GalNAc-T*^−/−^ mice and CST disruption in *CST*^−/−^ mice, *CST*^−/−^ × *GalNAc-T*^−/−^-Tg(neuronal) mice, *CST*^−/−^ × *GD3s*^−/−^ mice, and *CST*^−/−^ × *GalNAc-T*^−/−^ mice. The smaller band confirms GD3 synthase (GD3s) gene disruption in *CST*^−/−^ × *GD3s*^−/−^ mice. Additionally, a band identifying the GalNAc-T-flag in the *CST*^−/−^ × *GalNAc-T*^−/−^-Tg(neuronal) mouse strain confirms selective GalNAc-T gene reexpression neuronally. Confirmation of absence or selective expression of lipids was assessed in all genotypes with two anti-ganglioside antibodies that detected either the *a*- or *b*-series complex gangliosides and an anti-sulfatide antibody. Qualitative results for immunostaining with these antibodies in the peripheral nerve are shown in Figure 1*C*. As expected, neural tissue from all genotypes except *GalNAc-T*^−/−^ mice and *CST*^−/−^ × *GalNAc-T*^−/−^ mice was positive for anti-GM1 (*a*-series) antibody immunolabeling. Additionally, anti-GD1b antibody (*b*-series) binding was undetectable in *GalNAc-T*^−/−^ mice and *CST*^−/−^ × *GalNAc-T*^−/−^ genotypes, confirming the absence of all *a*- and *b*-series gangliosides. Similarly, nerves from *CST*^−/−^ × *GD3s*^−/−^ mice, which are null for *b*-series ganglioside expression, had no observable immunolabeling with the anti-GD1b antibody. Both *a*- and *b*-series gangliosides were successfully reintroduced in the axons of the *CST*^−/−^ × *GalNAc-T*^−/−^-Tg(neuronal) mouse line, as shown by positive anti-GM1 and anti-GD1b immunoreactivity. When neural tissue was immunostained with anti-sulfatide antibody, positively labeled myelin was observed in WT and *GalNAc-T*^−/−^ mice nerves, whereas no labeling was detected in the other four genotypes, confirming the absence of sulfatide expression with CST gene knock-out.

**Table 1.** Comparison of lipid and enzyme expression profiles among the mouse genotypes used in this study

Life expectancy	GalNAc-T enzyme expressed globally	CST enzyme expressed globally	Axonal complex ganglioside expression		Global *a*-series overexpression	Non-neuronal GM3 overexpression	Non-neuronal GD3 overexpression
			*a*-series	*b*-series			
WT	✓	✓	✓	✓	×	×	×
*GalNAc-T*^−/−^	×	✓	×	×	×	✓	✓
*CST*^−/−^	✓	×	✓	✓	×	×	×
*CST*^−/−^ × *GalNAc-T*^−/−^-Tg (neuronal)	×	×	✓	✓	×	✓	✓
*CST*^−/−^ × *GD3s*^−/−^	✓	×	✓	×	✓	×	×
*CST*^−/−^ × *GalNAc-T*^−/−^-Tg(glial)	×	×	×	×	×	✓	✓
*CST*^−/−^ × *GalNAc-T*^−/−^	×	×	×	×	×	✓	✓

### Ganglioside and sulfatide double-null mice have a significantly reduced lifespan

**Figure 2.**
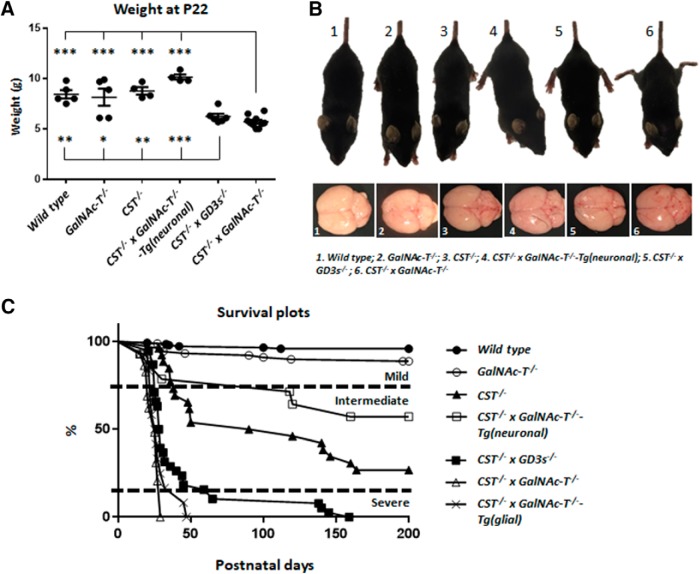
Altering the expression of gangliosides and sulfatide in novel transgenic mouse lines effects survival and phenotype. ***A***, Weight of double-null (*n* = 10) and *CST*^−/−^ × *GD3s*^−/−^ (*n* = 6) mice is normal during development, but declines from P15–P25 and is significantly reduced compared with other genotypes at P22. Statistical differences among genotypes were determined by one-way ANOVA followed by Tukey's *post hoc* tests to compare multiple comparisons, indicated on the graphs as follows: *p < 0.05; **p < 0.01; ***p < 0.001. ***B***, *CST*^−/−^ × *GalNAc-T*^−/−^, and *CST*^−/−^ × *GD3s*^−/−^ mice display a reduction in stature, hindlimb leg splaying, a hunched appearance, and tremor at P22. Gross brain anatomy does not differ between genotypes. ***C***, Survival plots demonstrate that incrementally diminishing ganglioside and sulfatide expression corresponds to a reduction in life expectancy of up to 200 d. WT (*n* = 7) and GalNAc-T^−/−^ (*n* = 10) mice have a normal life expectancy. In the absence of sulfatide with normal ganglioside expression, life expectancy is more than halved in *CST*^−/−^ mice (*n* = 18). Interestingly, in the absence of sulfatide and complex gangliosides with the reintroduction of complex gangliosides into neurons alone (*n* = 6), life expectancy is improved, with 60% of mice surviving to 200 d and beyond. Mice with no sulfatide and *a*-series gangliosides expressed globally (*n* = 38) can survive up to 20 weeks. Double ganglioside and sulfatide knock-out mice (*n* = 29) have the worst phenotype, never surviving past 4 weeks and dying at P21–P25. Reintroduction of gangliosides into glia (*n* = 12) does not improve survival.

The complex ganglioside-null and sulfatide-null mouse strains have an age-dependent degenerative phenotype that manifests clinically from 4 months and 6 weeks of age, respectively. In contrast to each of the single-glycolipid-deficient mice, the *CST*^−/−^ × *GalNAc-T*^−/−^ mice exhibited a very severe phenotype from 2 weeks of age, which was lethal by 4 weeks. The mice appear to develop normally and are similar in size to WT and single knock-out mice but then subsequently fail to thrive, declining in body weight beyond P15 and showing a significant reduction in weight at P22 (one-way ANOVA *F*_(5,28)_ = 18.15, *p* < 0.0001; Fig. 2*A*). Single pup rearing through removal of littermates on three occasions did not prolong survival. *CST*^−/−^ × *GalNAc-T*^−/−^ mice exhibit a hunched, emaciated appearance; develop a tremor; and display hind-limb leg splaying (Fig. 2*B*), a typical characteristic of the single knock-out mice at later stages of life. Despite reduction in weight, macroscopic brain (Fig. 2*B*) and nerve anatomy appeared comparable to other genotypes. Survival plots demonstrate that incrementally diminishing ganglioside and sulfatide expression corresponds with a reduction in life expectancy, measured up to 200 d (Fig. 2*C*).WT and *GalNAc-T*^−/−^ mice have a normal life expectancy up to 200 d. *CST*^−/−^ × *GalNAc-T*^−/−^ mice have the shortest life expectancy of the lines studied, never surviving beyond 4 weeks, with the majority only surviving to P21–P25. In the absence of sulfatide expression, but with normal ganglioside expression, *CST*^−/−^ life expectancy at 200 d more than halves. In mice lacking sulfatide and complex gangliosides globally but with all complex gangliosides reintroduced specifically into neurons [i.e., the *CST*^−/−^ × *GalNAc-T*^−/−^-Tg(neuronal) mouse], life expectancy is restored to that of sulfatide-null mice, with 60% of mice surviving to at least 200 d. Additionally, weight at P22 is comparable in this genotype to WT, *CST*^−/−^, and *GalNAc-T*^−/−^ mice (Fig. 2*A*). To determine whether *a*- or *b*-series complex ganglioside deficiency was responsible for the severity of the double sulfatide and complex ganglioside-null mouse, we examined mice that expressed *a*-series gangliosides only and lacked *b*-series gangliosides (achieved through crossing sulfatide- and *b*-series-null mice). These were only modestly improved relative to the *CST*^−/−^ × *GalNAc-T*^−/−^ phenotype, as shown by the failure to thrive and significant weight loss at P22 (Fig. 2*A*). Life expectancy is severely reduced, with only 50% survival at 4 weeks, and <10% mice reaching a maximum of 21 weeks (Fig. 2*C*). To determine the importance of neuronal relative to glial ganglioside expression, *CST*^−/−^ × *GalNAc-T*^−/−^ mice were generated with glial expression of complex gangliosides: *CST*^−/−^ × *GalNAc-T*^−/−^-Tg(glial). Unlike the successful rescue of the lethal phenotype with neuronal complex gangliosides, expression of complex gangliosides in glial membranes did not improve *CST*^−/−^ × *GalNAc-T*^−/−^ survival. Therefore based on survival data, the genotypes can be categorised into three subsets: mild (WT, *GalNAc-T*^−/−^); moderate (*CST*^−/−^, *CST*^−/−^ × *GalNAc-T*^−/−^-Tg(neuronal); and severe (*CST*^−/−^ × *GD3s*^−/−^, *CST*^−/−^ × *GalNAc-T*^−/−^) (Fig. 2*C*).

### Loss of both sulfatide and complex gangliosides results in a disruption to CNS NoRs

**Figure 3.**
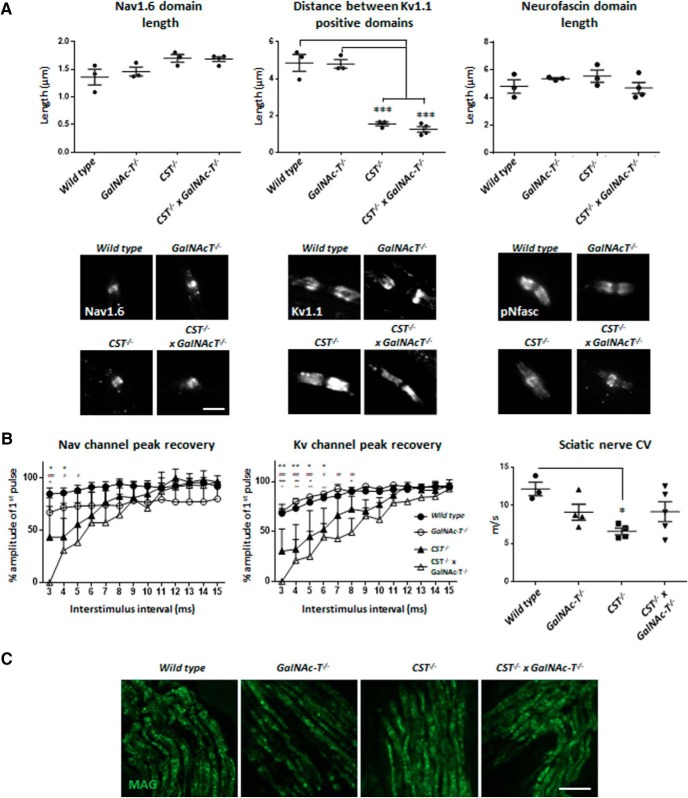
The additional loss of complex gangliosides on a sulfatide-null background does not augment disorganization at the NoR or electrophysiological function in the peripheral nervous system. ***A***, There is modest Nav1.6 channel cluster domain lengthening in both *CST*^−/−^ and *CST*^−/−^ × *GalNAc-T*^−/−^ teased SNs that does not reach significance. Representative images from teased SNs immunostained for Nav1.6 show that Nav channel cluster appearance was similar in every genotype. Disturbance of the paranode is indicated by invasion of Kv1.1 channels into the paranode, shown by a decrease in the gap between positive domains. The gap between Kv1.1-positive domains is significantly and comparably decreased in both *CST*^−/−^ and *CST*^−/−^ × *GalNAc-T*^−/−^ mice compared with WT and GalNAc-T^−/−^ NoRs. Representative images show that Kv1.1 formed two distinct domains of immunostaining at the juxtaparanodes in WT and *GalNAcT*^−/−^ mice. Conversely, mice lacking sulfatide expression had Kv1.1 staining at the paranodes, suggesting disruption to the axo–glial junction. The length of pNFasc-immunostained domains does not significantly differ among genotypes. However, representative images show that labeling greatly differs: WT and *GalNAc-T*^−/−^ NoRs have pNFasc staining that is most intense at the paranodes, forming two clear dimers (“normal”), whereas both *CST*^−/−^ and *CST*^−/−^ × *GalNAc-T*^−/−^ mice have intense staining at the nodal gap (presumed NF186) with weaker paranodal (presumed NF155) staining (“abnormal”), which indicates a disturbance in paranodal adhesion (WT, *n* = 3; *GalNAc-T*^−/−^, *n* = 3; *CST*^−/−^, *n* = 3; and *CST*^−/−^ × *GalNAc-T*^−/−^, *n* = 4). Scale bar, 5 μm. ***B***, Perineural recordings of Nav and Kv channel currents from intercostal nerves showing an increase in recovery time for both peaks following paired pulse stimulation in both *CST*^−/−^ and *CST*^−/−^ × *GalNAc-T*^−/−^ mice compared with WT and *GalNAc-T*^−/−^ mice. The *CST*^−/−^ and *CST*^−/−^ × *GalNAc-T*^−/−^ mice were not significantly different from each other at any ISI. Graphs display the means and ±SEM for each genotype (WT *n* = 5, *GalNAc-T*^−/−^ *n* = 2, *CST*^−/−^ *n* = 3, *CST*^−/−^ × *GalNAc-T*^−/−^ *n* = 2) and statistical analysis performed (two-way ANOVA; #WT vs *CST*^−/−^ × *GalNAc-T*^−/−^ mice; *WT vs *CST*^−/−^; +*GalNAc-T*^−/−^ vs *CST*^−/−^ × *GalNAc-T*^−/−^; ∧*GalNAc-T*^−/−^ vs *CST*^−/−^ × *GalNAc-T*^−/−^). SN conduction velocity decreased in *CST*^−/−^ (*n* = 4), *GalNAc-T*^−/−^ (*n* = 4), and *CST*^−/−^ × *GalNAc-T*^−/−^ (*n* = 5) mice compared with WT (*n* = 3), but only reached significance in *CST*^−/−^ nerves. ***C***, Peripheral nerve MAG immunostaining was comparable among genotypes. Scale bar, 20 μm. Graphs display the means and ±SEM for each genotype and statistical differences among genotypes were determined by one-way ANOVA followed by Tukey's *post hoc* tests to compare multiple comparisons, indicated on the graphs as follows: *p < 0.05; **p < 0.01; ***p < 0.001.

**Figure 4.**
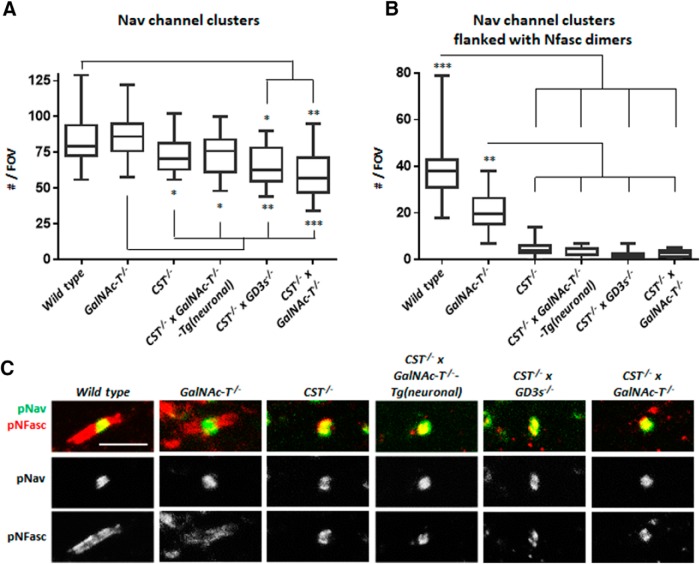
Loss of both sulfatide and complex gangliosides results in a modest reduction in CNS Nav channel cluster number and sulfatide deficiency reduces NF155 presence at paranodal loops. ***A***, The number of Nav channel clusters significantly decreases in glycolipid deficient mouse OpNs (*n* = 3–4/genotype). Reintroducing *a*- and *b*-series gangliosides into neurons rescues this feature. ***B***, Nav channel clusters flanked by normal paranodal NF dimers are significantly reduced in number when sulfatide is not expressed (*n* = 2–3/genotype). Box-and-whisker plots are used to display the spread of all data points collected from each animal and means were calculated per genotype for statistical analysis (one-way ANOVA followed by Tukey's *post hoc* tests to compare multiple comparisons, indicated on the graphs as follows: *p < 0.05; **p < 0.01; ***p < 0.001). ***C***, Representative images of OpN sections from each genotype double-immunostained for pan-Nav (pNav) antibody (green) and pNFasc antibody (red). Na channel clusters were observed in every genotype. pNFasc immunostaining formed a long band crossing the node and paranodes in WT and *GalNAc-T*^−/−^ mice, suggesting labeling of the NF186 and NF155 isoforms, respectively. All of the genotypes lacking sulfatide expression had pNFasc staining restricted to the NoR and colocalizing only with pNav staining, suggesting the presence of only the NF186 isoform of NF. Scale bar, 5 μm.

**Figure 5.**
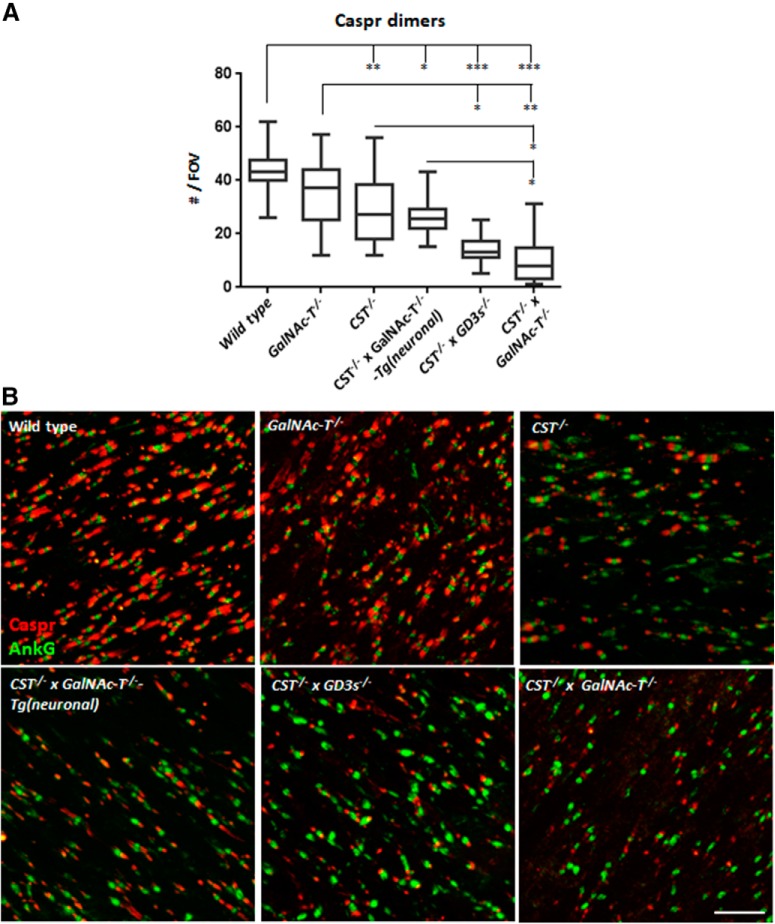
Paranodal Caspr dimer immunostaining in CNS tissue is progressively reduced with increasing glycolipid deficiency. ***A***, *CST*^−/−^ × *GalNAc-T*^−/−^ and *CST*^−/−^ × *GD3s*^−/−^ mice have significantly fewer Caspr dimers per FOV compared with WT and *GalNAc-T*^−/−^ mice and are not significantly different from each other (*n* = 2–5/genotype). Caspr dimer number is improved to levels within *GalNAc-T*^−/−^ mice range, but not WT, in *CST*^−/−^ and *CST*^−/−^ × *GalNAc-T*^−/−^-Tg(neuronal) mice, which both display significantly more Caspr dimers than *CST*^−/−^ × *GalNAc-T*^−/−^ mice. Box-and-whisker plots are used to display the spread of all data points collected from each animal and means were calculated per genotype for statistical analysis (one-way ANOVA followed by Tukey's *post hoc* tests to compare multiple comparisons, indicated on the graphs as follows: *p < 0.05; **p < 0.01; ***p < 0.001). ***B***, Representative images of OpN sections from each genotype double-immunostained for Caspr (red) and the nodal marker AnkyrinG (green) showing the reduction in Caspr dimer number flanking ankyrin G clusters with diminishing glycolipid expression. Scale bar, 10 μm.

Age-dependent loss of nodal integrity is evident in both single ganglioside- and sulfatide-null mice and could potentially contribute to loss of normal ion channel clustering, functional deficits, and axon degeneration. Of the single-lipid-deficient mice, disrupted nodal integrity is more rapid and pronounced in the sulfatide-null mice beginning after myelin development (>1 month). To assess whether this process was enhanced or accelerated in *CST*^−/−^ × *GalNAc-T*^−/−^ mice, we initially assessed changes in both the PNS (SN, internal intercostal nerve) and the CNS (OpN, brain) at P22. When the PNS integrity was examined, we found that there was no change in number of nodes per ROI among genotypes (one-way ANOVA, *F*_(3,9)_ = 0.1261, *p* = 0.94; WT = 4.07 ± 1.09; *GalNAc-T*^−/−^ = 4.27 ± 0.97; *CST*^−/−^ = 3.9 ± 0.15; *CST*^−/−^ × *GalNAc-T*^−/−^ = 4.5 ± 0.58) and therefore focused on characterizing changes to nodal protein immunostaining. In general, combined sulfatide and complex ganglioside deficiency did not augment the disorganization of proteins at the NoR of the single sulfatide-null mice (Fig. 3*A*). Peripheral nerve Nav channel cluster domains were lengthened both in *CST*^−/−^ and *CST*^−/−^ × *GalNAc-T*^−/−^ SNs. Despite results reaching significance (one-way ANOVA, *F*_(3,9)_ = 0.5794, *p* = 0.0491), *post hoc* multiple comparisons showed the Nav channel cluster domain length in the latter genotypes did not reach significance compared with WT, *GalNAc-T*^−/−^, or each other. In both *CST*^−/−^ and *CST*^−/−^ × *GalNAc-T*^−/−^ SN NoRs, immunostaining for Kv1.1 channel domains normally located at the juxtaparanodes invaded the paranodal region, shown by a shortening in the distance between the staining domains, compared with WT and *GalNAc-T*^−/−^ nerves (one-way ANOVA, *F*_(3,9)_, *p* < 0.0001). This indicated impairment to paranodal organization. Despite the fact that pNFasc staining length did not significantly change among genotypes (one-way ANOVA, *F*_(3,9)_ = 1.157, *p* = 0.38), the overall appearance of the staining pattern did differ. WT and GalNAc-T^−/−^ NoRs have pNFasc staining that is most intense at the paranodes forming two clear dimers (normal), whereas both the CST^−/−^ and CST^−/−^ × GalNAc-T^−/−^ mice have intense staining at the nodal gap (the NF186 component) with weaker paranodal (the NF155 component) staining (abnormal). By undertaking qualitative assessment of these “normal” and “abnormal” staining patterns, we found that WT and GalNAc-T^−/−^ mice had significantly more normal pNFasc immunostaining than CST^−/−^ and CST^−/−^ × GalNAc-T^−/−^ mice, which were similar to each other (two-way ANOVA, *F*_(3,18)_ = 9.56, *p* = 0.0063; WT = 88% ± 2.8; GalNAc-T^−/−^ = 75.67% ± 4.5; CST^−/−^ = 29.67% ± 3.2; CST^−/−^ × GalNAc-T^−/−^ = 35% ± 10.2). These results, along with the evident Kv1.1 invasion of the paranodal region, indicate a disturbance in paranodal organization. Additionally, electrophysiological function did not significantly worsen in *CST*^−/−^ × *GalNAc-T*^−/−^ peripheral nerves compared with single sulfatide-deficient nerve based on paired pulse stimulations and conduction velocity measurements (Fig. 3*B*). We performed perineural recordings to assess the Nav and Kv channel currents of the internal intercostal nerve that innervates the traingularis sterni muscle. The recovery of both peaks after paired pulse stimulation was significantly different among genotypes (Nav peak: two-way ANOVA, *F*_(3,117)_ = 10.79, *p* < 0.0001. Kv peak: two-way ANOVA, *F*_(3,117)_ = 32.24, *p* < 0.0001). Both *CST*^−/−^ and *CST*^−/−^ × *GalNAc-T*^−/−^ peaks were significantly reduced in the initial ISIs compared with WT and *GalNAc-T*^−/−^ preparations, but did not differ from on**e** another, as indicated on the graphs in Figure 3*B*. Conduction velocity was slower in *GalNAc-T*^−/−^, *CST*^−/−^, and *CST*^−/−^ × *GalNAc-T*^−/−^ SN compared with WT, but only reached significance in *CST*^−/−^ nerve (one-way ANOVA, *F*_(3,12)_ = 3.939, *p* = 0.036). MAG immunostaining was comparable in peripheral nerves of all four genotypes (Fig. 3*C*). It is thus unlikely that PNS dysfunction and degeneration would account for the lethal phenotype in double-deficient mice and there was no evidence of further disruption to the PNS in the double-deficient mice compared with the sulfatide-null mice. Therefore, we then focused our attention on the CNS with additional Tg lines. When comparing Nav channel clustering in the OpN from all genotypes, there is a significant reduction in Nav channel cluster number (one-way ANOVA (*F*_(5,13)_ = 4.76, *p* = 0.0108). *CST*^−/−^ × *GalNAc-T*^−/−^ mice have fewer Nav channel clusters than all other genotypes, but this reduction only reached significance compared with WT and complex-ganglioside-deficient mice, which were not significantly different to each other (Fig. 4*A*). Although reduced, the number of Nav channel clusters is within the normal WT range when sulfatide is expressed or complex gangliosides are reintroduced in the neuronal membrane in *CST*^−/−^ × *GalNAc-T*^−/−^-Tg(neuronal) mice. This is not the case when *a*-series gangliosides are expressed globally on the sulfatide-null background in *CST*^−/−^ × *GD3s*^−/−^ mice, which have similar cluster numbers to *CST*^−/−^ × *GalNAc-T*^−/−^ mice. Because cluster number does not explicitly define mature NoRs, the numbers of Nav channel clusters flanked laterally by intact paranodal NF dimers were assessed. Earlier reports have shown that lack of sulfatide attenuates NF155 localization to the paranodal region (Ishibashi et al., 2002; Schafer et al., 2004; Marcus et al., 2006) and, indeed, we found that all four lines with sulfatide deficiency had significantly fewer NF-positive paranodal dimers flanking Nav channel clusters as early as P22 compared with WT and *GalNAc-T*^−/−^ mice (one-way ANOVA *F*_(5,13)_ = 21.31, *p* < 0.0001) (Fig. 4*B*,*C*). A pNFasc antibody was used that detects both the neuronal NF186 and glial NF155 isoforms critical for Nav channel clustering and paranodal axo–glial junction formation, respectively. We detected no change to presumed nodal NF186 presence and observed normal colocalization with Nav channel clusters, suggesting that this isoform is not affected by ganglioside or sulfatide lipid expression. Further analysis showed that paranodal Caspr dimers flanking Ankyrin G were significantly reduced at P22 in all genotypes, with the exception of *GalNAc-T*^−/−^ mice, compared with WT mice (one-way ANOVA (*F*_(5,14)_ = 8.8, *p* = 0.0006) (Fig. 5). However, significance was greatest when WT was compared with *GalNAc-T*^−/−^ × *CST*^−/−^ and *CST*^−/−^ × *GD3s*^−/−^ mice. *GalNAc-T*^−/−^ × *CST*^−/−^ mice also showed a significant reduction compared with *CST*^−/−^ and *CST*^−/−^ × *GalNAc-T*^−/−^-Tg(neuronal) nerves. Expression of neuronal *a*- and *b*-series gangliosides improved the feature under examination, but global *a*-series ganglioside expression did not. Our results show a more exaggerated loss in paranodal NF than Caspr, as judged by immunostaining. We attribute this finding to normal localization during early development, with subsequent loss of NF155 followed by Caspr as the maturing node begins to deteriorate over time. However, it is also possible that the pNFasc antibody is not as sensitive as the Caspr antibody, accounting for apparent relative quantitative differences.

### Glycolipid deficiency compromises CNS axon integrity and function

**Figure 6.**
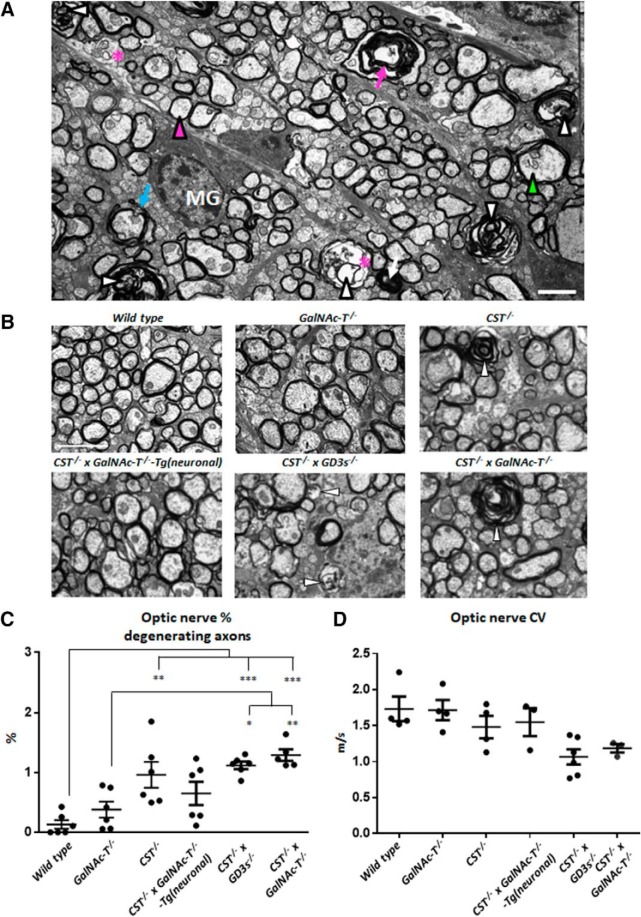
Glycolipid deficiency causes pathological changes and compromises CNS axon survival and function. ***A***, Representative ultrastructural features observed in *CST*^−/−^ × *GD3s*^−/−^ and *CST*^−/−^ × *GalNAc-T*^−/−^ mice. Shown are normal myelinated axon (magenta arrowhead), degenerating axons (white arrowheads), dark condensed degenerating axon (white arrow), vacuoles within axons (*), empty myelin sheath (magenta arrow), redundant myelin (blue arrow), abnormal cytoskeleton (green arrowhead), and microglia (MG). ***B***, Electron micrographs depicting the differences among the genotypes. White arrowheads indicate degenerating axons. Myelin is similar among mouse lines. ***C***, Degenerating axon number increases to a significant level compared with WT in OpNs from *CST*^−/−^, *CST*^−/−^ × *GD3s*^−/−^, and *CST*^−/−^ × *GalNAc-T*^−/−^ mice. Reintroducing *a*- and *b*-series gangliosides into neurons rescues this pathology. ***D***, Reduction in conduction velocity was observed in OpNs with diminishing glycolipid content. This did not reach significance between genotypes. One-way ANOVA followed by Tukey's *post hoc* tests to compare multiple comparisons, indicated on the graphs as follows: *p < 0.05; **p < 0.01; ***p < 0.001. Scale bar, 2 μm.

Age-dependent degeneration beyond 2 months of age is a feature of the *CST*^−/−^ and the *GalNAc-T*^−/−^ mouse strains. The principal ultrastructural abnormality observed in the current study was early onset CNS axon degeneration in the *CST*^−/−^ × *GalNAc-T*^−/−^ mice characterized by axons with dark condensed cytoplasm, organelle or vacuole filled axons, or empty myelin sheaths with or without axonal fragments. We have indicated the various abnormalities observed in OpNs from *CST*^−/−^ × *GalNAc-T*^−/−^ mice in a representative image (Fig. 6*A*). Electron micrographs indicate degenerating axons (arrowheads) and show the normal formation of myelin in large-diameter myelinated fibers, alongside abundant unmyelinated and thinly myelinated fibers undergoing myelin maturation in all genotypes (Fig. 6*B*). There was a significant increase in degenerating axons in the OpN at P25 with diminishing glycolipid expression (one-way ANOVA (*F*_(5,29)_ = 9.408, *p* < 0.0001). *CST*^−/−^ × *GalNAc-T*^−/−^ mice had significantly more degenerating axons compared with WT and *GalNAc-T*^−/−^ nerves, which are comparable (Fig. 6*C*). The number of degenerating axons in *CST*^−/−^ mice was very variable and reached significance compared with WT. Additionally, the number of degenerating axons in *CST*^−/−^ mice was lower than that observed for *CST*^−/−^ × *GalNAc-T*^−/−^ OpN, but this difference did not reach significance. Protection from degeneration in axons was achieved by reinstatement of both *a*- and *b*-series gangliosides neuronally, leading to a reduction in the number of degenerating axons to within WT and *GalNAc-T*^−/−^ levels. However, the number of degenerating axons was not improved by expression of global *a*-series ganglioside expression in the *CST*^−/−^ × *GD3s*^−/−^ strain, which were equal to *CST*^−/−^ × *GalNAc-T*^−/−^ levels. To investigate the functional consequence of degenerating axons and nodal structure disturbance, conduction velocity was recorded from isolated OpNs at P22 (Fig. 6*D*). Conduction velocity was significantly reduced with diminishing glycolipid expression (one-way ANOVA *F*_(5,18)_ = 4.25, *p* = 0.01), which correlated with the survival plots and nodal and ultrastructural data. The reduction in conduction velocity did not reach significance between any of the genotypes in *post hoc* analysis.

### MAG protein expression in myelin is disrupted in sulfatide and ganglioside double-null mice

**Figure 7.**
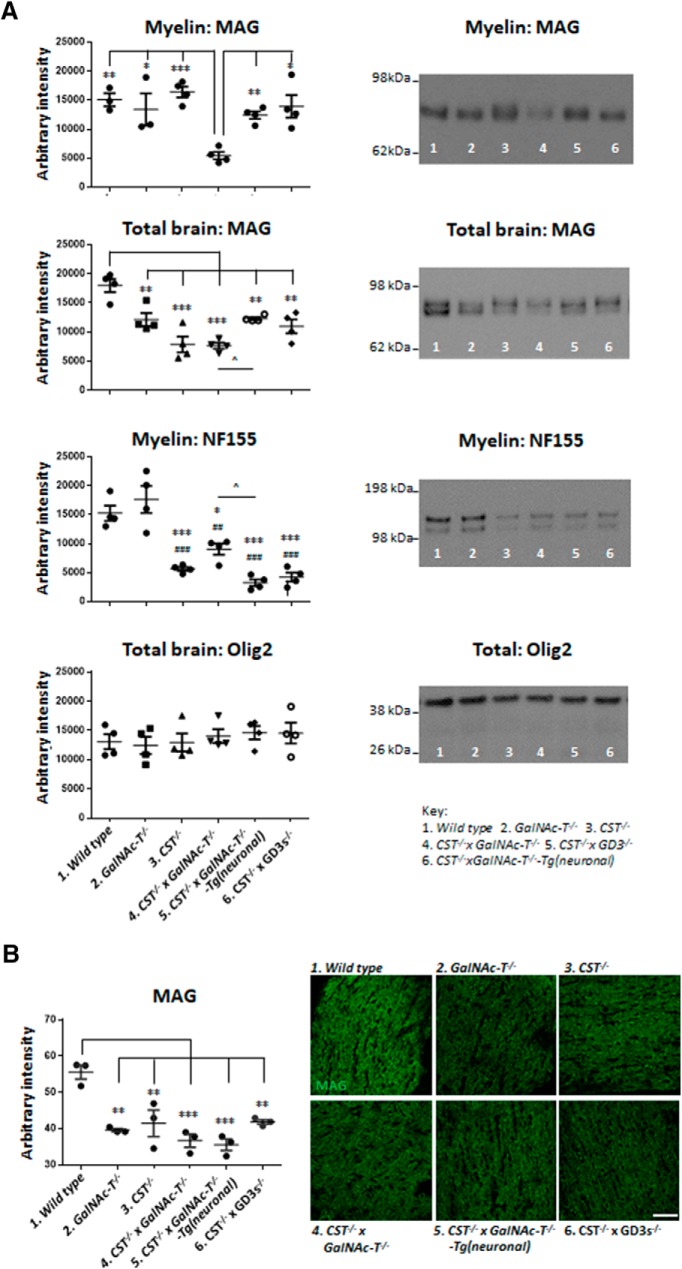
MAG and NF155 expression in the myelin fraction is altered by glycolipid deficiency. Note the new order of genotypes compared to other figures. ***A***, In the myelin fraction from P22 brain homogenates, MAG is significantly reduced in *CST*^−/−^ × *GalNAc-T*^−/−^ mice compared with all genotypes, which do not significantly differ from one another. All genotypes have a significant reduction in MAG expression in whole-brain homogenate compared with WT. Myelin NF155 was significantly reduced in all genotypes compared with WT (*) and *GalNAc-T*^−/−^ (#), which had similar levels. ∧Significant difference between *CST*^−/−^ × *GalNAc-T*^−/−^ and *CST*^−/−^ × *GalNAc-T*^−/−^-Tg(neuronal) mice. Olig2 is unchanged by altered glycolipid expression. Representative blots to the right of corresponding graphs show protein intensity per genotype, as indicated by the corresponding number in the key below. ***B***, P22 OpN MAG immunostaining is significantly reduced in all genotypes compared with WT nerves, which do not differ significantly from one another. Representative images show MAG immunostaining per genotype. Means were calculated per genotype for statistical analysis (one-way ANOVA followed by Tukey's *post hoc* tests to compare multiple comparisons, indicated on the graphs as follows: *p < 0.05; **p < 0.01; ***p < 0.001). Scale bar, 50 μm. ^##^*p* < 0.01, ^###^*p* < 0.001, ^∧^*p* < 0.05.

Sulfatide deficiency has been reported previously to alter the expression of key myelin proteins, prompting us to interrogate differences in the expression of these proteins in the six genotypes studied. Expression of the major myelin proteins PLP and MBP in the myelin fraction from P22 brain homogenates did not significantly differ among the six genotypes, with the exception of a mildly reduced expression in WT mice compared with *GalNAc-T*^−/−^ genotypes (data not shown). A significant reduction in MAG protein level was observed in the myelin fraction from *CST*^−/−^ × *GalNAc-T*^−/−^ mice compared with all genotypes, which did not significantly differ from one another (one-way ANOVA *F*_(5,16)_ = 7.735, *p* = 0.0007) (Fig. 7*A*). Conversely, MAG expression in whole-brain homogenates and in OpN sections was comparably reduced in all genotypes compared with WT, suggesting an enhanced loss of appropriate trafficking or site-specific anchoring in myelin in the *CST*^−/−^ × *GalNAc-T*^−/−^ mice (WB: one-way ANOVA *F*_(5,18)_ = 13.87, *p* < 0.0001; immunostaining one-way ANOVA *F*_(5,12)_ = 13.36, *p* = 0.0001) (Fig. 7*A*,*B*). This notion is strengthened by the comparable expression of oligodendrocyte transcription factor 2 (Olig2) among genotypes, which indicates oligodendrocyte number is normal and oligodendrocytes are not lost due to glycolipid deficiency (one-way ANOVA *F*_(5,12)_ = 0.41, *p* = 0.835) (Fig. 7*A*). Myelin NF155 protein levels were significantly reduced to a comparable level in all genotypes deficient in sulfatide, which corresponds with our immunostaining data and previous reports (one-way ANOVA *F*_(5,18)_ = 23.41, *p* < 0.0001) (Fig. 7*A*). This was replicated in the whole-brain extract (data not shown).

## Discussion

Glycosphingolipids, including sulfatide and complex gangliosides, have a significant role in myelinated nerve maintenance, but their distinct or interdependent roles and interactions are unclear. The neurodegenerative consequence of deficiency in either sulfatides or complex ganglioside synthesis through transgenic manipulation indicates a role for these lipids in maintaining myelin and axonal integrity, particularly at the NoR (Takamiya et al., 1996; Sheikh et al., 1999; Honke et al., 2002; Ishibashi et al., 2002; Marcus et al., 2002; Hoshi et al., 2007; Susuki et al., 2007). It was the aim of this study to investigate their interdependency on interacting membranes to add insight into the role that each family of lipids plays in myelinated nerve and nodal stability.

We found that interbreeding sulfatide-deficient mice with complex-ganglioside-deficient mice resulted in an exaggerated neurodegenerative phenotype and early death compared with single-null mice. Through a combination of ultrastructural, immunohistological, functional, and biochemical studies, we showed that the combined loss of both groups of lipids resulted in a more severe outcome than single lipid deficiency (Takamiya et al., 1996; Honke et al., 2002). Diminishing expression of lipids divided the survival of the seven genotypes produced for this study into three broad groups: mild (WT and *GalNAc-T*^−/−^), intermediate [*CST*^−/−^ and *CST*^−/−^ × *GalNAc-T*^−/−^-Tg(neuronal)], and severe [*CST*^−/−^ × *GD3s*^−/−^, *CST*^−/−^ × *GalNAc-T*^−/−^-Tg(glial), and *CST*^−/−^ × *GalNAc-T*^−/−^]. We reported a more pronounced phenotype in the CNS than the PNS and thus focused much of our analysis on the CNS. Degenerating axon number increased with diminishing lipid content and conduction became increasingly impaired; MAG expression in the myelin fraction from brain homogenates was significantly lower in the *CST*^−/−^ × *GalNAc-T*^−/−^ genotype compared with all other genotypes and Nav channel cluster number, Caspr dimer number, and Nav channels flanked by NF155 dimers decreased with decreasing lipid expression. Rescuing the neuronal, but not the glial, complex ganglioside expression reversed the lethality observed in the double-null mice. Examining the relative importance of *a*- and *b*-series gangliosides, we observed that only modest improvement occurred with global *a*-series ganglioside expression on a sulfatide-null background. Collectively, these data indicate the importance of neuronal *b*-series gangliosides (e.g., GD1b and GT1b) in maintaining survival in the co-presence of sulfatide deficiency and indicate interdependency between the functions of these two groups of lipids. We thus propose that sulfatide and *b*-series ganglioside lipid domains on opposing membranes majorly contribute to a coordinated axo–glial adhesion and paranodal organization, a combined loss of which leads to severe impairment of nerve integrity with a fatal outcome at an early age.

We have previously highlighted the significance of neuronal complex ganglioside expression to nerve integrity in the *GalNAc-T*^−/−^ age-dependent neurodegenerative genotype (Yao et al., 2014). Considering the impact of neuronal ganglioside expression on survival combined with the reduction of MAG in the myelin fraction in double-deficient mice, these findings suggest an underlying mechanism for the lethal phenotype in *CST*^−/−^ × *GalNAc-T*^−/−^ mice. The complex gangliosides GD1a and GT1b are prominently expressed on the axonal membrane and are receptors for MAG (Collins et al., 1997; Vinson et al., 2001). MAG is expressed on the periaxonal membrane of the myelin sheath (Bartsch et al., 1989; Trapp et al., 1989) and is involved in the continuous axo–glial contact and bidirectional signaling along the myelinated nerve, particularly at the paranode. MAG forms *cis*-bonds in the myelin membrane that are stabilized by *trans* interactions with gangliosides in the neuronal membrane (Pronker et al., 2016). MAG-null mice exhibit delayed maturation of nodes and, similar to *GalNAc-T*^−/−^ mice, modest nervous system abnormalities, suggesting a role in myelinated nerve maintenance rather than development (Montag et al., 1994; Li et al., 1998; Marcus et al., 2002; Pan et al., 2005). It has been shown previously that MAG levels are reduced in *GalNAc-T*^−/−^ mice (Sheikh et al., 1999; Kawai et al., 2001), which suggests that, when considered with the comparable phenotype between *GalNAc-T*^−/−^ and *MAG*^−/−^ mice, complex gangliosides and MAG cooperatively contribute to the stability of the axo–glial junction. Indeed, interbreeding *GalNAc-T*^−/−^ and *MAG*^−/−^ strains did not exacerbate the phenotype of the single-null mice, suggesting a complementary and functional interaction between these molecules (Pan et al., 2005). Conversely, in our study, combining *GalNAc-T*^−/−^ and *CST*^−/−^ strains exacerbated the phenotype, suggesting two independent roles. MAG acts as a myelin receptor for axonal gangliosides and could be localized to the myelin membrane by sulfatide-rich lipid rafts. Recent analysis of protein extracts from 4-week-old sulfatide-null mouse brains confirmed a progressive reduction in MAG and a significant reduction in NF155 (25% vs WT) (Palavicini et al., 2016). Together with extraction studies suggesting that sulfatide-containing lipid rafts could be anchors for MAG and NF155 (Pomicter et al., 2013), it seems that sulfatide may act as a wide-ranging anchor for multiple myelin and glial membrane proteins. With the knowledge that MAG and sulfatide modulate axo–glial stability, Marcus et al. (2002) investigated the significance of both molecules to axo–glial integrity by crossing MAG-deficient mice with GalC/sulfatide double-deficient mice, which individually have similar phenotypes. Similar to our lethal sulfatide and ganglioside double deficiency phenotype, this genetic combination resulted in a lethal phenotype, with survival up to P22 (Marcus et al., 2002). Again, the NoR appear to develop normally followed by subsequent generalized impairment of the paranodal axo–glial junction. Unlike the minimal effect of combined MAG and complex ganglioside disruption, MAG and ceramide galactosyltransferase double deficiency aggravates the phenotype, suggesting that two nonredundant functions are impaired. This phenotype corresponds temporally to our lethal phenotype, indicating that a loss of interaction between MAG and gangliosides in a membrane environment devoid of GalC and sulfatide is fatal. It is likely that GalC/sulfatide has a role in promoting localization/anchoring of MAG and, subsequently, MAG/ganglioside promote axo–glial integrity. Indeed, we reported a striking deficiency in MAG in the myelin fraction of our double-null mice compared with all other genotypes because of the loss of its glial membrane localizing agent, sulfatide, and its binding partner, complex gangliosides.

The severe neurodegenerative phenotype of our double-lipid-deficient mice reflected that of paranodal-protein-deficient mice (Tait et al., 2000; Bhat et al., 2001; Boyle et al., 2001; Charles et al., 2002). In the current study, we restricted our investigation of nodal disruption to those proteins related to function (Nav and Kv channels) or previously identified as lipid raft associated (NF155, Caspr) (Sheikh et al., 1999; Schafer et al., 2004; Susuki et al., 2007; Pomicter et al., 2013) and acknowledge that this was not an exhaustive study. Adhesion molecules freely diffuse in the axon membrane and accumulate at NoR, whereas ion channels and components of the cytoskeleton require transport (Zhang et al., 2012). NF186 is essential for maintenance of the Nav channel clusters at the NoR (Zhang et al., 2012; Desmazieres et al., 2014; Taylor et al., 2017) and appeared unperturbed in our double-null mice. This aligns with the fact that NF186 is likely not raft associated (Schafer et al., 2004; Susuki et al., 2007). The presence of NF186 accounts for the relatively minimal reduction in Na channel clusters and impairment in conduction observed in *CST*^−/−^ × *GalNAc-T*^−/−^ mice. Schafer et al. (2004) proposed an indirect mechanism of axo–glial junction stabilization at the paranode whereby glycosphingolipid-rich lipid rafts cluster NF155 that then co-cluster with axonal binding partners Caspr and contactin. Indeed, we confirmed by immunostaining and Western blot that NF155 was disrupted in all genotypes with sulfatide deficiency. The axonal partner of NF155, Caspr, was also disrupted, as had been shown previously in single ganglioside and sulfatide deficiency (Ishibashi et al., 2002; Susuki et al., 2007), but to a greater degree when both sets of lipids were absent. Caspr has been associated with ganglioside-rich lipid rafts on the axonal membrane and, in their absence, Caspr is not anchored (Susuki et al., 2007). Our results that the PNS was less perturbed than CNS likely reflect the greater stability of the peripheral NoR and the greater influence that the paranode has on stability in the CNS (Rasband and Peles, 2015). However, the combined absence of both NF155 and Caspr through loss of both sulfatide and gangliosides, respectively, likely intensifies the reduction in Nav channel clusters and ultimately leads to enhanced nodal instability compared with single-lipid-null mice.

Ganglioside, sulfatide, and MAG single-deficient mice do not exhibit a severe phenotype, strongly suggesting redundant functions: overexpression/loss of other lipids (e.g., simple gangliosides, seminolipid) could, in theory, contribute to the overall phenotype in transgenic mice. Mice that lack the GD3s enzyme that underlies production of all *b*-series gangliosides (Fig. 1*A*) have grossly normal nodal architecture (Okada et al., 2002), demonstrating that there is compensation in the ganglioside family. It is possible that ganglioside-deficient mice have a less severe phenotype than those lacking sulfatide due to compensation by overexpression of simple gangliosides, notably GM3 and GD3, the levels of which are markedly elevated in the *GalNAc-T*^−/−^ phenotype (Susuki et al., 2007). The increased severity of double-ganglioside-null (GM3-only) mice (Kawai et al., 2001; Inoue et al., 2002; Yamashita et al., 2005) lends support to this idea; however, the nodal protein organization has not been characterized. It was therefore an unexpected finding that the *CST*^−/−^ × *GD3s*^−/−^ strain had such a severe phenotype. This could be explained by the lack of the *b*-series ganglioside GT1b interacting with MAG, although these mice express large amounts of GD1a, another axonally localized MAG-binding ganglioside (Vinson et al., 2001).

**Figure 8.**
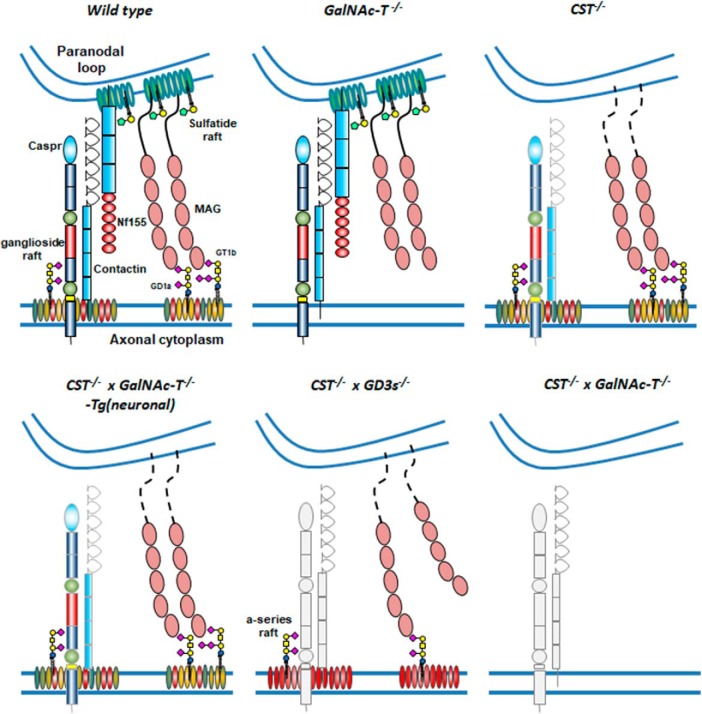
Schematic depicting the glycolipid rafts and their associated paranodal proteins Caspr, NF155, and MAG under WT conditions and in our transgenic mouse lines. GD1a and GT1b are represented in the ganglioside rafts because they are the major ligands of MAG; however, in reality, the rafts would contain all complex gangliosides. Normally, GD1a and GT1b in rafts will tether MAG, but, in their absence, MAG does not make the axo–glial connection. If NF155 is present, then this protein can partner with Caspr/contactin to make an axo–glial junction. However, when sulfatide is absent, NF155 is also lost from the paranode. In the absence of NF155, Caspr is also diminished, especially with loss of complex ganglioside rafts. Under conditions of both ganglioside and sulfatide deficiency, we propose an absence of the structurally supporting proteins MAG and NF155 that results in the loss of a functionally competent axo–glial junction.

It is clear from these results that sulfatide and complex gangliosides have crucial and independent roles in clustering proteins on opposing membranes that are essential to normal axo–glial integrity and nervous system function, as shown in Figure 8. Redundant features can compensate for the loss of one family, but their simultaneous absence leads to catastrophic axon degeneration, axo–glial disruption, and nodal pathology, culminating in early death.
